# Differential sensitivity of polyhydroxyalkanoate producing bacteria to fermentation inhibitors and comparison of polyhydroxybutyrate production from *Burkholderia cepacia* and *Pseudomonas pseudoflava*

**DOI:** 10.1186/1756-0500-6-219

**Published:** 2013-06-04

**Authors:** Diane Dietrich, Barbara Illman, Casey Crooks

**Affiliations:** 1 USDA Forest Service, Forest Products Laboratory, One Gifford Pinchot Drive, Madison, WI 53726, USA

**Keywords:** Polyhydroxyalkanoate, Fermentation inhibitors, Bioconversion

## Abstract

**Background:**

The aim of this study is determine the relative sensitivity of a panel of seven polyhydroxyalkanoate producing bacteria to a panel of seven lignocellulosic-derived fermentation inhibitors representing aliphatic acids, furans and phenolics. A further aim was to measure the polyhydroxybutyrate production of select organisms on lignocellulosic-derived monosaccharides arabinose, xylose, glucose and mannose.

**Findings:**

We examined the sensitivity of seven polyhydroxyalkanoate producing bacteria: *Azohydromonas lata*, *Bacillus megaterium*, *Bacillus cereus*, *Burkholderia cepacia*, *Pseudomonas olevorans*, *Pseudomonas pseudoflava* and *Ralstonia eutropha*, against seven fermentation inhibitors produced by the saccharification of lignocellulose: acetic acid, levulinic acid, coumaric acid, ferulic acid, syringaldehyde, furfural, and hyroxymethyfurfural. There was significant variation in the sensitivity of these microbes to representative phenolics ranging from 0.25-1.5 g/L coumaric and ferulic acid and between 0.5-6.0 g/L syringaldehyde. Inhibition ranged from 0.37-4 g/L and 0.75-6 g/L with acetic acid and levulinic acid, respectively. *B*. *cepacia* and *P*. *pseudoflava* were selected for further analysis of polyhydroxyalkanoate production.

**Conclusions:**

We find significant differences in sensitivity to the fermentation inhibitors tested and find these variations to be over a relevant concentration range given the concentrations of inhibitors typically found in lignocellulosic hydrolysates. Of the seven bacteria tested, *B*. *cepacia* demonstrated the greatest inhibitor tolerance. Similarly, of two organisms examined for polyhydroxybutyrate production, *B*. *cepacia* was notably more efficient when fermenting pentose substrates.

## Findings

### Background

Numerous bacteria have been shown to synthesize polyhydroxyalkanoates (PHAs) as intracellular carbon storage granules in response to nutrient limitations [1]. PHAs are a class of biopolyesters with potential to serve as replacements for petroleum based plastics and also have the additional benefits of being biodegradable, biocompatible, and exhibit greater UV-resistance than compounds such as polypropylene or polylactic acid biopolymers [2]. Currently, the costs of PHA production are prohibitive for use in broad-scale implementation. As the carbon fermentation substrate for PHA production represents approximately 50% of the cost, the development of inexpensive carbon sources would significantly reduce the overall cost of PHA production [3]. Lignocellulosic material from agricultural residues, forest thinnings, and pre-pulping hemicellulosic hydrolysates represent a potentially vast, inexpensive and renewable carbon source.

Saccharification of lignocellulosic material from the aforementioned potential reservoir of inexpensive carbon is complicated by the concomitant generation of a number of fermentation inhibitors [4,5]. These microbial fermentation inhibitors broadly consist of three classes that include furans, aliphatic acids, and phenolics. Furans such as furfural and hydroxymethylfurfural (HMF) are generated by the dehydration of pentoses and hexoses, respectively. Aliphatic acids such as levulinic and formic acid result from further degradation of these furan aldehydes, while acetic acid is released by hydrolysis during dilute acid pretreatment of lignocellulosic feedstock. Finally, lignin breakdown products result in a variety of toxic phenolic compounds related to the three monolignol precursors coniferyl, coumaryl and sinapyl alcohols.

Microorganisms vary considerably in their tolerance of, and their ability to catabolize, these inhibitors. A number of bacteria have been identified that are able to metabolize both phenolic compounds and furans and these traits may be useful in overcoming the barriers presented by these compounds in PHA bioproduction [6,7]. In this study, we examine a panel of PHA producing bacteria and assess their comparative tolerance to representatives of the three classes of lignocellulosic-derived fermentation inhibitors described. We further examine the PHA production efficiency of two of these microbes, *B*. *cepacia* and *P*. *pseudoflava*, selected for their known broad monosaccharaide utilization capabilities, and assess their PHA production efficiency using authentic lignocellulosic-derived sugars arabinose, xylose, glucose and mannose as fermentation substrates.

We find significant variation in both the tolerance of these microbes to the panel of fermentation inhibitors and in PHA production. While fermentation inhibitors can be removed by a variety of means including liming, membrane filtration, and adsorption to charcoal these methods add costs that undermine the effort to recover inexpensive carbon sources. Characterization of inhibitor tolerance and PHA production will allow a more informed basis for microbe selection and strain improvement for PHA production from lignocellulosic material, affording a biological approach to overcoming fermentation inhibitors.

### Results

We find a considerable range of sensitivity to microbial inhibitors in the bacteria tested. Furans as a class were the least toxic of the compounds on a w/v basis. The minimum inhibitory concentration (MIC) for furfural was 1.5 g/L (*A*. *lata*), 4 g/L (*P*. *pseudoflava*) and 6 g/L for all other organisms tested (Table 1). Only *A*. *lata* showed sensitivity to HMF within the scope of this assay at 6 g/L while all other organisms had tolerances exceeding 6 g/L. These levels are generally below furan concentrations found in a number of lignocellulose hydrolysates indicating this class may be comparatively insignificant as a barrier to efficient PHA production as concentrations of these inhibitors in lignocellulosic hydrolysates is generally observed to be below 2 g/L [Crooks, unpublished observations]. Our results differ from Pan and colleagues who observed complete inhibition of *B*. *cepacia* at 1 g/L furfural [8]. The discrepancy between results may be due to different media or incubation methods used and warrants further investigation.

**Table 1:** **Minimum inhibitory concentrations** (**g**/**L**) **of representative fermentation inhibitors**^***a***^

**Bacteria**	**AA**	**LA**	**CA**	**FA**	**syr**	**HMF**	**fur**
*Azohydromonas lata*	2.00	4.00	0.38	0.38	1.00	6.00	1.50
*Bacillus megaterium*	0.75	1.50	0.38	0.38	1.50	>6.00	6.00
*Burkholderia cepacia*	4.00	6.00	1.50	1.50	6.00	>6.00	6.00
*Bacillus cereus*	1.50	3.00	0.38	0.38	4.00	>6.00	6.00
*Pseudomonas olevorans*	0.75	1.50	0.38	0.75	6.00	>6.00	6.00
*Pseudomonas pseudoflava*	0.38	0.75	0.25	0.25	0.50	>6.00	4.00
*Ralstonia eutropha*	2.00	4.00	0.38	0.75	2.00	>6.00	6.00

Data represents the mean of quadruplicate samples with values >90% growth inhibition (OD_600_) reported.

^*a*^Abbreviations: *AA* acetic acid, *LA* levulinic acid, *CA* coumaric acid, *FA* ferulic acid, *syr* syringaldehyde, *HMF* hydroxymethylfurfural, *fur* furfural.

Coumaryl and coniferyl-derived phenolic representatives were the most toxic on a w/v basis. Coumaric acid and ferulic acid MIC values ranged from 0.25 g/L (*P*. *pseudoflava*) to 1.5 g/L (*B*. *cepacia*) (Table 1). The syringyl-derived representative syringaldyhyde was comparatively less toxic on a w/v basis with MIC values ranging from 0.5 g/L (*P*. *pseudoflava*) to 6.0 g/L (*B*. *cepacia and P*. *olevorans*).

Aliphatic organic acids were intermediate in toxicity on a w/v basis with acetic acid MIC values ranging from 0.38 g/L (*P*. *pseudoflava*) to 4 g/L *B*. *cepacia*) and levulinic acid MIC values ranging from 0.75 g/L (*P*. *pseudoflava*) to 4.0 g/L (*B*. *cepacia*) (Table 1).

Within each inhibitor class, *B*. *cepacia* exhibited the greatest inhibitor tolerance of the organisms tested. With the exception of *A*. *lata* sensitivity to furfural, *P*. *pseudoflava* was consistently the most sensitive organism in these assays. These data indicate that *B*. *cepacia* represents a promising candidate for lignocellulosic bioconversion when considering tolerance to fermentation inhibitors.

**Figure 1:**
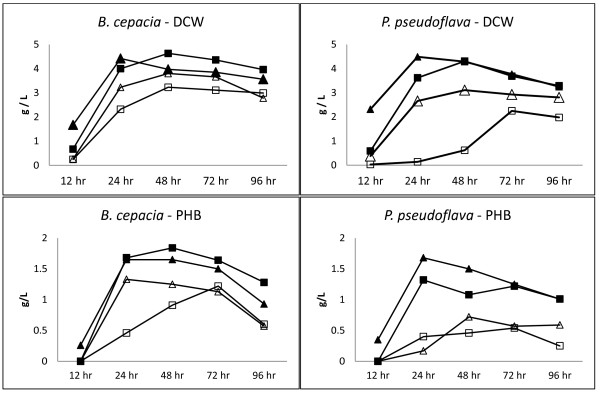
**Bacterial growth and PHB production from *B. cepacia *and *P. pseudoflava. ***Dry cell weight (DCW, top) and PHB production (bottom) for *B*. *cepacia* (left) and *P*. *pseudoflava* (right) was determined on glucose (closed triangles), mannose (closed squares), xylose (open triangles) and arabinose (open squares). Data represents the mean of duplicate samples.

Recognizing the broad monosaccharide utilization capabilities of *B*. *cepacia* and *P*. *pseudoflava*, we examined the PHB production potential of these two bacteria in chemically defined media supplemented with 10 g/L of glucose, mannose, arabinose or xylose. Both organisms achieved higher dry cell weight (DCW) on hexose substrates glucose and mannose compared to pentose substrates arabinose and xylose (Figure 1, top). Similarly, PHB production was more efficient using hexose substrates. *B*. *cepacia* and *P*. *pseudoflava* were comparable in DCW formation and PHB production on hexose substrates glucose and mannose (Figure 1, bottom). However, with pentose substrates *B*. *cepacia* exhibited more rapid growth and PHB production than *P*. *pseudoflava*. Specifically, *B*. *cepacia* DCW was 1557% and 21% greater at 24 hours, and 421% and 22% greater at 48 hours on arabinose and xylose, respectively (Figure 1). Similarly, PHB production with *B*. *cepacia* was 15% and 98% greater at 24 hours, and 682% and 74% greater at 48 hours arabinose and xylose, respectively (Figure 1).

This study revealed significant variations in tolerance to fermentation inhibitors between the seven PHA-producing bacteria at concentrations relevant to those observed in lignocellulosic hydrolysates. *Burkholderia cepacia* was the most resistant to the phenolic, aliphatic organic acid, and furan compounds tested and also yielded the greatest PHA production of the two bacteria tested. *B*. *cepacia* has been shown to efficiently convert xylose to PHB and to also produce the more valuable polyhydroxybuyrate-co-valerate copolymer when supplemented with the cosubstrate levulinic acid [9]. In this work we show that *B*. *cepacia* can also utilize the lignocellulose derived carbohydrates mannose and arabinose in addition to xylose and glucose as substrates for PHA production. The higher inhibitor tolerance, broad substrate utilization, and copolymer producing capacity of *B*. *cepacia* make it an excellent candidate for continued development for PHA production from lignocellulosic biomass.

### Methods

#### Bacterial strains and media

*Azohydromonas lata* ATCC 29714, *Bacillus cereus* ATCC 14579, *Bacillus megaterium* ATCC 14581, *Burkholderia cepacia* ATCC 17759, *Pseudomonas olevorans* ATCC 29347, *Pseudomonas pseudoflava* ATCC 33668 and *Ralstonia eutropha* ATCC 17699 were used in this study. All bacteria were maintained and propagated at 30°C on NBY media consisting of nutrient broth (Difco 231000) supplemented with 1 g/L yeast extract, and 15 g/L agar as appropriate.

#### Inhibitor assay

Minimal inhibitory concentrations (MICs) were determined by standard methods [10]. MICs were scored at the dilution point where 90% inhibition of increase in optical density at OD_600_ nm was observed. Briefly, 80 g/L stocks of the indicated inhibitors were prepared in DMSO and sterilized by 0.2 μm filtration. Serial dilutions were performed in 96 well plates containing a 1:50 dilution of overnight cultures of the indicated bacteria suspended in 1/2x NBY media using initial inhibitor concentrations of 6 g/L and 4 g/L generating parallel overlapping serial dilution profiles. Plates were incubated at 30°C and 85% relative humidity and analyzed at 24 hours.

#### PHA production

Shake flask cultures for PHA production were performed essentially as described [11]. Minimal salts media containing (per liter) 6.7 g Na_2_HPO_4_^.^ 7H_2_O, 1.5 g KH_2_PO_4,_ 60 μl 10% (w/v) ferric ammonium citrate, 1.5 g (NH_4_)_2_SO_4,_ 0.5 g CaCl_2_^.^ 2H_2_O, 1 g MgSO_4_^.^ 7H_2_O and 1 ml trace element solution. The trace element solution contained (per liter): 300 mg H_3_BO_,_ 200 mg CoCl_2_^.^ 6H_2_O, 100 mg ZnSO_4_^.^ 7H_2_O, 30 mg MnCl_2_^.^ 4H_2_O, 30 mg NaMoO_4_^.^ 2H_2_O, 20 mg NiCl_2_^.^ 6H_2_O and 10 mg CuSO_4_^.^ 5H_2_O. Filter sterilized (0.2 μm) carbon sources were added as indicated. Culture volumes were 200 ml in 2 L Erlenmeyer flasks and agitated at 200 rpm on 1 inch orbits at 30°C. Samples for analysis were collected at 12, 24, 48, 72 and 96 hours (Figure 1).

#### PHA analysis

10 ml samples of PHA-producing shake flask cultures were collected at the indicated times, pelleted by centrifugation at 5 kg^-1^ for 20 min at 16°C and dried overnight at 100°C. Samples were weighed and methyl ester derivatization was performed essentially as described [12]. 3 ml of 15% (v/v) methanol/H_2_SO_4_ were added to samples followed by 3 ml CHCl_3_ and incubated at 100°C for 4 hr. 1.5 ml dH_2_0 was added after which the samples were vortexed vigorously. The chloroform layer was recovered and 2 μl was injected using a HP5890A equipped with a 7683 auto sampler, split-splitless capillary column injection port, a Flame Ionization Detector and a 35 m Agilent DB-Wax capillary column. The temperature of the injector was 230°C and the detector temperature 275°C. The oven program used was 80°C for 4 minutes, then ramping 8°C/min to 160°C, for 6 minutes for a total run time of 20 minutes. Helium was used as the carrier at 20 ml/min.

## Competing interests

The authors declare they have no competing interests. This work was performed at and financially supported by the USDA – Forest Service, Forest Products Laboratory, Madison WI.

## Authors’ contributions

DD performed sample preparation and GC analysis. BI provided experimental concepts and edited the manuscript. CC designed the experiments, performed MIC assays and fermentations, and wrote the manuscript. All authors read and approved the final manuscript.
